# Structure-guided covalent stabilization of coronavirus spike glycoprotein trimers in the closed conformation

**DOI:** 10.1038/s41594-020-0483-8

**Published:** 2020-08-04

**Authors:** Matthew McCallum, Alexandra C. Walls, John E. Bowen, Davide Corti, David Veesler

**Affiliations:** 1 Department of Biochemistry, University of Washington, Seattle, WA USA; 2 Humabs Biomed SA, a Subsidiary of Vir Biotechnology, Bellinzona, Switzerland

**Keywords:** SARS-CoV-2, Cryoelectron microscopy

## Abstract

SARS-CoV-2 is the causative agent of the COVID-19 pandemic, with 10 million infections and more than 500,000 fatalities by June 2020. To initiate infection, the SARS-CoV-2 spike (S) glycoprotein promotes attachment to the host cell surface and fusion of the viral and host membranes. Prefusion SARS-CoV-2 S is the main target of neutralizing antibodies and the focus of vaccine design. However, its limited stability and conformational dynamics are limiting factors for developing countermeasures against this virus. We report here the design of a construct corresponding to the prefusion SARS-CoV-2 S ectodomain trimer, covalently stabilized by a disulfide bond in the closed conformation. Structural and antigenicity analyses show we successfully shut S in the closed state without otherwise altering its architecture. We demonstrate that this strategy is applicable to other β-coronaviruses, such as SARS-CoV and MERS-CoV, and might become an important tool for structural biology, serology, vaccine design and immunology studies.

## Abstract (web summary)

The conformational dynamics of SARS-CoV-2 spike are constrained by engineering a disulfide bond that locks the protein in a closed conformation, a strategy that was also applied to SARS-CoV and MERS-CoV.

## Main

In the past two decades, three zoonotic coronaviruses crossed the species barrier to cause severe pneumonia in humans: (1) severe acute respiratory syndrome coronavirus (SARS-CoV), which was associated with an epidemic in 2002–2003 and a few additional cases in 2004 (refs. ^1,2^); (2) Middle-East respiratory syndrome coronavirus (MERS-CoV), which is currently circulating in the Arabian peninsula^3^; and (3) SARS-CoV-2, the etiological agent of the ongoing COVID-19 pandemic^4,5^. SARS-CoV-2 was discovered in December 2019 in Wuhan, Hubei Province of China, was sequenced and isolated by January 2020 (refs. ^4,6^) and has infected over 11 million people with more than 532,000 fatalities as of 5 July 2020. No vaccines or specific therapeutics are licensed to treat or prevent infections from any of the seven human-infecting coronaviruses with the exception of remdesivir^7,8^, which was recently approved by the Food and Drug Administration for emergency use for COVID-19 treatment.

**Fig. 1:**
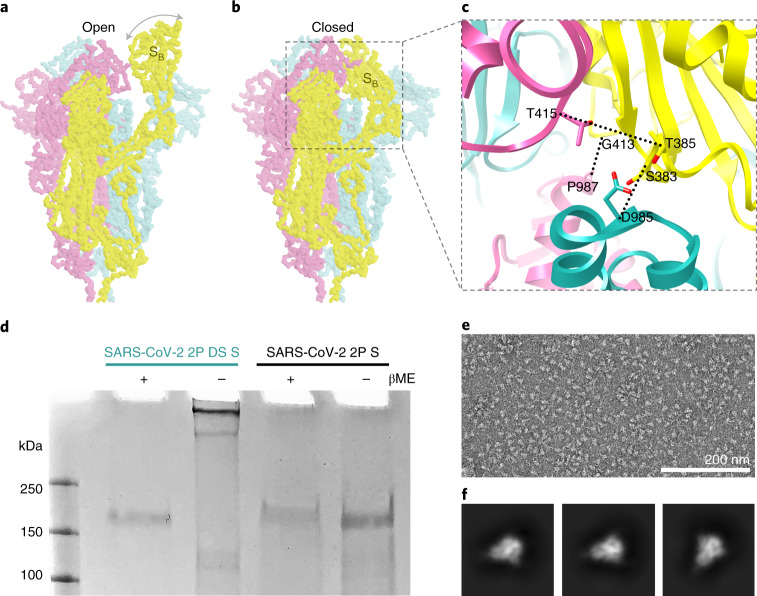
Structure-based engineering of a SARS-CoV-2 S trimer in the closed conformation. **a**,**b**, Cryo-EM structures of SARS-CoV-2 S with one S^B^ receptor-binding domain open (**a**, PDB 6VYB) and one in the closed state (**b**, PDB 6VXX), used as a basis for the design of intermolecular disulfide bonds^18^. **c**, Pairs of residues mutated to create potential disulfide bonds are shown with dashed black lines between the Cα In panels **a**–**c**, each S protomer is colored distinctly. **d**, SDS–PAGE analysis in reducing and nonreducing conditions showing formation of an intermolecular disulfide bond. βME, β-mercaptoethanol. The uncropped image is shown in Supplementary Data 1. **e**,**f**, Electron micrograph of negatively stained SARS-CoV-2 2P DS S confirming proper folding of the designed protein construct (**e**) and representative two-dimensional class averages (**f**).

Coronaviruses gain access to host cells using the homotrimeric transmembrane S glycoprotein protruding from the viral surface^9^. S comprises two functional subunits: S_1_ (encompassing the A, B, C and D domains) and S_2_. These subunits are responsible for binding to the host cell receptor and fusion of the viral and cellular membranes, respectively^10^. For many coronaviruses, including the newly emerged SARS-CoV-2, S is cleaved at the boundary between the S_1_ and S_2_ subunits which remain noncovalently bound in the prefusion conformation^10–18^. The distal S_1_ subunit comprises the receptor-binding domain(s), and contributes to stabilization of the prefusion state of the membrane-anchored S_2_ subunit which contains the fusion machinery^10,17,19–25^. Structural fluctuations of the receptor-binding S^B^ domain (also known as RBD), from a closed to an open conformation, enable exposure of the receptor-binding motif (RBM) which mediates interaction with angiotensin-converting enzyme 2 (ACE2) for SARS-CoV-2 (refs. ^6,18,26–31^) and SARS-CoV^32,33^, or dipeptidyl-peptidase 4 for MERS-CoV^34,35^ (Fig. 1a,b). Receptor engagement or interaction with the Fab fragment of the S230 neutralizing monoclonal antibody were previously shown to induce the SARS-CoV S cascade of conformational changes leading to membrane fusion, which we proposed to proceed through a molecular ratcheting mechanism^22,36^. For all coronaviruses, upon receptor binding S is further cleaved by host proteases at the S_2_′ site located immediately upstream of the fusion peptide^14,16,37^. This cleavage has been proposed to activate the protein for membrane fusion via extensive irreversible conformational changes^13–16,19,38,39^. As a result, coronavirus entry into susceptible cells is a complex process that requires the concerted action of receptor binding and proteolytic processing of the S protein to promote virus–cell fusion.

Viral fusion proteins, including coronavirus S glycoproteins, fold in a high-energy, kinetically trapped prefusion conformation found at the viral surface before host cell invasion^40^. This metastable state is activated with exquisite spatial and temporal precision upon encounter of a target host cell by one or multiple stimuli such as pH change^41,42^, proteolytic activation^13,15^ or protein–protein interactions^43^. The ensuing irreversible and large-scale structural changes of viral fusion proteins are coupled to fusion of the viral and host membrane to initiate infection. As a result, the postfusion state of a viral fusion protein is the lowest energy conformation (that is, ground state) observed throughout the reaction coordinates^40^. A notable exception to this general pathway is the vesicular stomatitis virus fusion glycoprotein G that can reversibly transition from the prefusion to the postfusion conformations and vice versa^42,44,45^.

The intrinsic metastability of viral fusion proteins—which is oftentimes magnified by working with ectodomain constructs lacking the transmembrane and cytoplasmic segments—has posed challenges for studying the structure and function of these glycoproteins as well as for vaccine design. As a result, a variety of approaches have been implemented to stabilize these fragile glycoproteins. Proline substitutions preventing refolding to an elongated α-helical structure observed in postfusion influenza virus hemagglutinin were reported as a promising strategy to stabilize the prefusion state of this widely studied viral glycoprotein^46^. Engineering approaches based on this concept along with introduction of designed disulfide bonds and other mutations have subsequently been used for stabilizing the prefusion conformation of other class I fusion proteins, such as the SOSIP mutations in the HIV-1 envelope glycoprotein^47–50^. Structure-guided prefusion stabilization via introduction of disulfide bonds and cavity-filling mutations was successfully implemented for the respiratory syncytial virus fusion glycoprotein^51^ (DS-Cav1) and parainfluenza virus 1–4 fusion glycoproteins^52^. Designed disulfide bonds have also proven useful to enhance the prefusion stability of the Hendra virus fusion glycoprotein^53^, mutations which were later applied to the Nipah virus fusion protein^54^. Finally, the introduction of double proline substitutions, herein 2P, to prevent fusogenic conformational changes of MERS-CoV S (ref. ^20^) and SARS-CoV S (ref. ^55^) was shown to stabilize the prefusion states of these glycoproteins. These results provided proof of concept of the broad applicability of this approach to coronavirus S glycoproteins, which was subsequently confirmed by its successful use for SARS-CoV-2 S structural studies^18,56,57^. In spite of these advances, the conformational dynamics and limited stability of the SARS-CoV-2, SARS-CoV and MERS-CoV S glycoproteins remain a challenge that needs to be overcome to accelerate structural studies of the immune response elicited by coronavirus infections and vaccine design. Recent reports of the observation of postfusion trimers at the surface of purified authentic SARS-CoV-2 (refs. ^58–60^) and of spontaneous refolding of a fraction of S trimers upon detergent-solubilization^61^ showcase these limitations.

We report here the design of a prefusion-stabilized SARS-CoV-2 S ectodomain trimer construct engineered to remain in the closed conformation through introduction of an intermolecular disulfide bond. Single-particle cryo-EM analysis of this glycoprotein coupled with ELISA assays unambiguously demonstrated that our strategy successfully shut S in the closed state without otherwise altering its architecture, as evaluated by binding to a panel of human monoclonal neutralizing antibodies and a COVID-19 convalescent serum. We show that this covalent stabilization strategy enhances the SARS-CoV-2 S resistance to proteolysis and that it is applicable to other β-coronavirus S glycoproteins. We envisage that it might become an important tool for vaccine design, structural biology, serology and immunology studies.

## Results

### Stabilizing SARS-CoV-2 S in the closed conformation

We reasoned that arresting the first step of SARS-CoV-2 S refolding—the S^B^ transition from a closed to an open conformation (Fig. 1a,b)—might enhance the stability of the prefusion state and yield a useful molecular tool. We therefore set out to engineer SARS-CoV-2 S stalled in the closed conformation through introduction of disulfide bonds into the ectodomain construct we previously used to determine structures of the closed and open conformations^18,57^. Specifically, our construct harbored an abrogated furin S_1_/S_2_ cleavage site (R682S, R683G and R685G)^10,24,36,62^, two consecutive proline stabilizing mutations (K986P and V987P, so called 2P)^20,55^ and a C-terminal foldon trimerization domain^63^. We designed the following pairs of cysteine substitutions aimed at introducing three intermolecular disulfide bonds per trimer: S383C/D985C, G413C/P987C and T385C/T415C (Fig. 1c). S383C/D985C was identified using the Disulphide by Design web-server^64^, while G413C/P987C and T385C/T415C were selected manually.

Of the three pairs of substitutions tested, only the S383C/D985C (termed SARS-CoV-2 2P DS S; DS, disulfide) could be recombinantly expressed using HEK293 Freestyle cells and purified. The yield is about tenfold reduced compared with SARS-CoV-2 2P S, presumably due to introduction of an intermolecular disulfide bond. SDS–PAGE analysis of SARS-CoV-2 2P DS S in reducing and nonreducing conditions demonstrated that the engineered disulfide bond was indeed correctly introduced (Fig. 1d). Further characterization of purified SARS-CoV-2 2P DS S using negative-staining electron microscopy indicated proper homotrimer folding and assembly (Fig. 1e,f).

### Structure of SARS-CoV-2 S stabilized in the closed conformation

**Extended Data Fig. 1:**
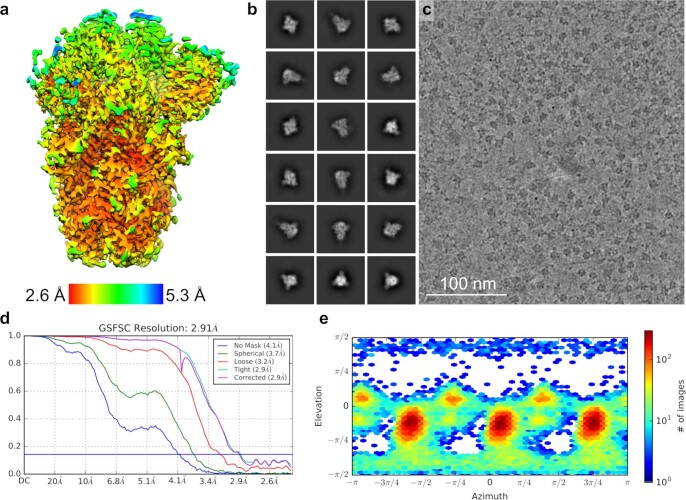
CryoEM data processing and validation. **a**. Local resolution map calculated using cryoSPARC. **b-c**. Representative electron micrograph (c) and class averages (b) of SARS-CoV-2 2P DS S embedded in vitreous ice. Scale bar: 100 nm. **d**. Gold-standard Fourier shell correlation curves. The 0.143 cutoff is indicated by an horizontal blue line. **e**. Particle orientation distribution plot.

**Extended Data Fig. 2:**
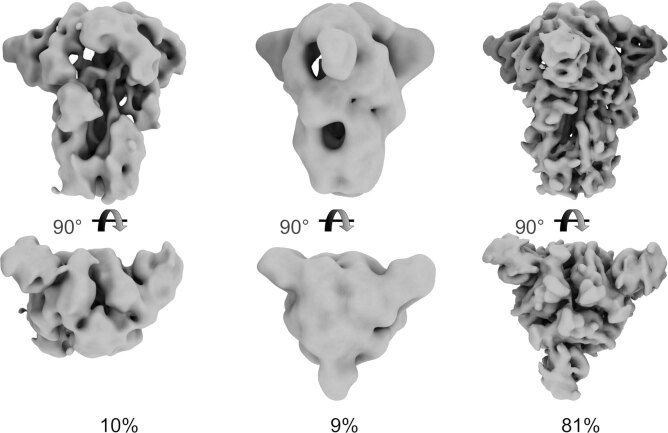
Orthogonal views of the classes obtained by 3D classification. Percentages reflect the proportion of particles that classified with each map.

Since the DS substitutions connect regions of the S glycoprotein that are far apart upon S^B^ receptor-binding domain opening and transition to the postfusion S state^19,36^, we expected this protein construct to be trapped in the closed S state via molecular stapling. To validate our design strategy, we used single-particle cryo-EM to analyze the conformational landscape of SARS-CoV-2 2P DS S (Table 1 and Extended Data Fig. 1). Three-dimensional (3D) classification of the cryo-EM dataset demonstrated that all particle images clustered in 3D reconstructions of the closed S trimer (Extended Data Fig. 2). In contrast, about half of the particle images selected from our previous SARS-CoV-2 2P S apo dataset corresponded to the closed S trimer, whereas the other half was accounted for by a partially open S trimer^18^. These results therefore indicate that we successfully engineered a shut closed S trimer.

**Table 1:** Cryo-EM data collection, refinement and validation statistics

	SARS-CoV-2 DS S (EMD-22083, PDB 6X79)
**Data collection and processing**	
Magnification (nominal)	36,000
Voltage (kV)	200
Electron exposure (e^–^ Å^−2^)	60
Defocus range (μm)	0.8–3.0
Pixel size (Å)	1.16
Symmetry imposed	*C*3
Initial particle images (no.)	100,295
Final particle images (no.)	46,181
Map resolution (Å)	2.9
FSC threshold	0.143
Map resolution range (Å)	2.6–5
**Refinement**	
Initial model used	PDB 6VXX
Model resolution (Å)	3.2
FSC threshold	0.5
Map sharpening *B* factor (Å^2^)	−74
Model composition	
Nonhydrogen atoms	45,816
Protein residues	2,850
Ligands	51
* B* factors (Å^2^)	
Protein	27.67
Ligand	26.9
R.m.s. deviations	
Bond lengths (Å)	0.013
Bond angles (°)	1.210
**Validation**	
MolProbity score	0.67
Clashscore	0.18
Poor rotamers (%)	0.26
Ramachandran plot	
Favored (%)	97.54
Allowed (%)	2.46
Disallowed (%)	0
EMRinger score	3.18

**Fig. 2:**
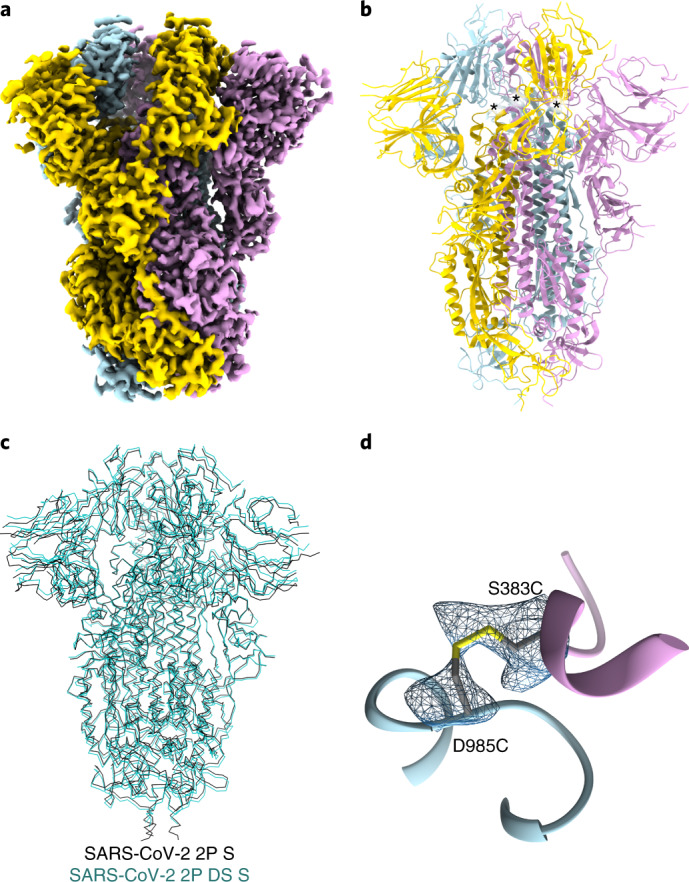
Cryo-EM structure of the closed SARS-CoV-2 2P DS S glycoprotein. **a**, Cryo-EM map of the SARS-CoV-2 2P DS S trimer in the closed conformation at 2.9-Å resolution. **b**, Ribbon diagram of the SARS-CoV-2 2P DS S trimer atomic model in the same orientation as in panel **a**. In panels **a** and **b**, each S protomer is colored distinctly. Asterisks show the locations of the introduced disulfide bonds. **c**, Superimposition of the SARS-CoV-2 2P DS S trimer (green) to the coordinates from the 2.8-Å SARS-CoV-2 2P S structure in the closed conformation, PDB 6VXX (ref. ^18^) (black). **d**, Enlarged view of the designed disulfide bond with the corresponding region of cryo-EM density shown as a blue mesh.

**Extended Data Fig. 3:**
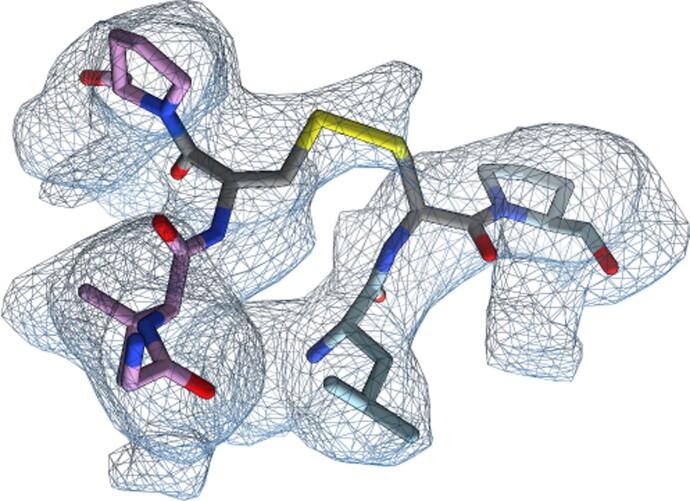
Cryo-EM structure of the closed SARS-CoV-2 DS S glycoprotein. Zoomed-in view of the designed disulfide bond with the corresponding region of cryo-EM density shown as a blue mesh.

We subsequently determined a 3D reconstruction of SARS-CoV-2 2P DS S at 2.9-Å resolution (applying threefold symmetry) (Fig. 2a,b). The cryo-EM map shows a good agreement with our previously determined structure in the closed conformation^18^; their respective models could be superimposed with a Cα r.m.s. deviation of 1.37 Å over 946 aligned residues (Fig. 2c). The cryo-EM density also resolves the disulfide bond between an S^B^ receptor-binding domain residue facing towards the fusion machinery (S383C) and the hairpin preceding the S_2_ subunit central helix (D985C) from a neighboring protomer (Fig. 2d and Extended Data Fig. 3), the latter residue being located directly upstream from the K986P and V987P prefusion-stabilizing mutations. These findings not only validate the structure-based design strategy but also show that it did not induce distortions of the S trimer. We note that density at the C-terminal stem helix was not resolved in SARS-CoV-2 2P DS S—accounting for six amino acid residues—whereas this region was visible in previously reported apo S maps^18,56^. This region was also absent from the SARS-CoV-2 S/S309 neutralizing antibody complex map^57^.

### Evaluation of SARS-CoV-2 2P DS S antigenicity

**Fig. 3:**
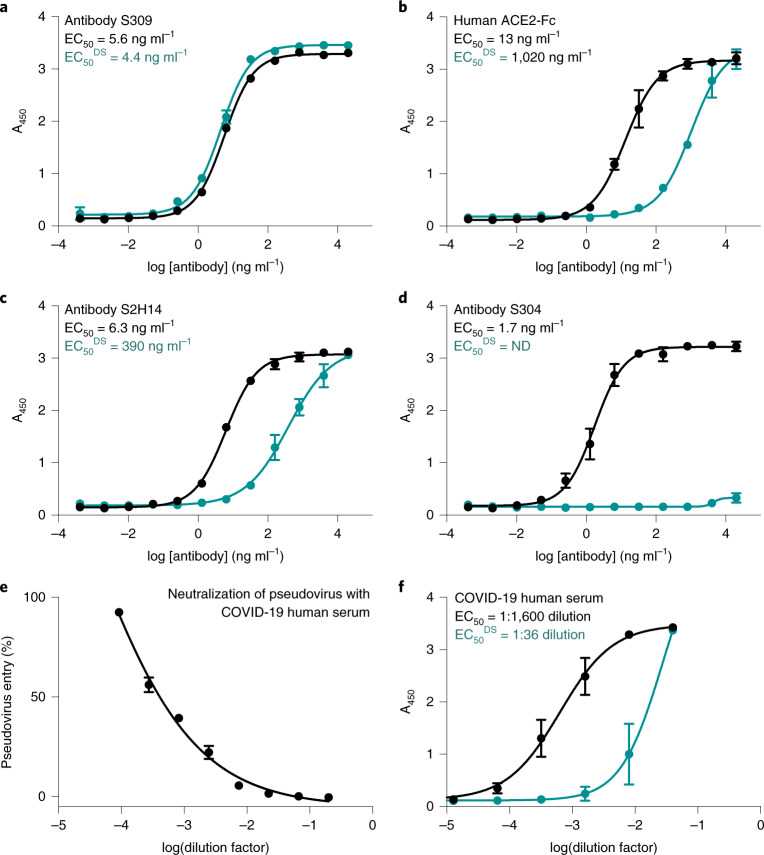
Evaluation of SARS-CoV-2 2P DS S antigenicity. **a**–**d**, Binding to immobilized SARS-CoV-2 2P DS S (green) or SARS-CoV-2 2P S (black) of serially diluted concentrations of the human neutralizing antibodies S309 (**a**), S2H14 (**c**) and S304 (**d**) and the human ACE2 receptor fused to human Fc (**b**). **e**, Neutralization of SARS-CoV-2 S pseudovirus with human serum obtained from a patient with COVID-19. **f**, Binding of a serial dilution of the neutralizing convalescent serum shown in panel **e** to immobilized SARS-CoV-2 2P DS S (green) or SARS-CoV-2 2P S (black). Data are shown as mean and s.d. of *n* = 2 technical replicates; data are representative of two independent experiments. Data behind all graphs are available in Supplementary Data 1. A_450_, absorbance at 450 nm; ND, not determined.

The high structural similarity between the SARS-CoV-2 2P DS S structure presented here and our previously reported SARS-CoV-2 2P S structure (in the closed conformation)^18^ led us to hypothesize that they would have similar antigenicity profiles. To probe the influence of the introduced disulfide bond on antigenicity, we evaluated binding of SARS-CoV-2 2P DS S and SARS-CoV-2 2P S, side-by-side, to a panel of human neutralizing antibodies by ELISA. Binding to S309 was indistinguishable between the two constructs (Fig. 3a), in agreement with the fact that this antibody recognizes an epitope within the S^B^ receptor-binding domain that remains accessible in both the open and closed states^57^.

Given that the RBM is concealed in the closed conformation, we hypothesized that SARS-CoV-2 2P DS S binding to ACE2 and RBM-targeted antibodies would be hindered. As expected, ELISA experiments showed that SARS-CoV-2 2P DS S recognized ACE2 with two-orders-of-magnitude-reduced binding response compared with SARS-CoV-2 2P S (Fig. 3b). Furthermore, the binding response of the RBM-targeted S2H14 antibody to SARS-CoV-2 2P DS S was also dampened by two orders of magnitude relative to SARS-CoV-2 2P S, due to conformational masking of the RBM in the closed conformation^18^ (Fig. 3c). Although the S304 antibody interacted with SARS-CoV-2 2P S in a concentration-dependent manner, it did not bind to SARS-CoV-2 2P DS S (Fig. 3d). Since we previously demonstrated that S304 recognizes an epitope distinct from both the RBM and the S309 epitope, it is expected that this antibody binds to a cryptic epitope that is only accessible upon S^B^ opening, as is the case for the CR3022 antibody^65–68^. Collectively, these findings validate that SARS-CoV-2 2P DS S is in a native closed conformation and illustrate the usefulness of this protein construct to investigate epitopes recognized by neutralizing antibodies.

We subsequently assessed binding of the serum from a patient convalescing from COVID-19 to SARS-CoV-2 2P DS S and SARS-CoV-2 2P S by ELISA. The sample was obtained from a Washington State donor who had a high serum antibody neutralization titer (Fig. 3e). Comparison of half-maximal binding titers showed that recognition of SARS-CoV-2 2P DS S was ~40-fold weaker than that of SARS-CoV-2 2P S (Fig. 3f). This difference likely reflects the proportion of antibodies directed to the RBM or cryptic epitopes similar to the one recognized by S304 in this serum sample. As SARS-CoV-2 2P DS S only displays closed S^B^ receptor-binding domains within the context of a folded trimer, we suggest it will be a useful tool for serology studies aiming at evaluating antibody responses in patients with COVID-19, and could complement tests using ACE2 inhibition as a proxy for evaluating the presence of neutralizing antibody titers in the human population.

### Evaluation of SARS-CoV-2 2P DS S stability

**Fig. 4:**
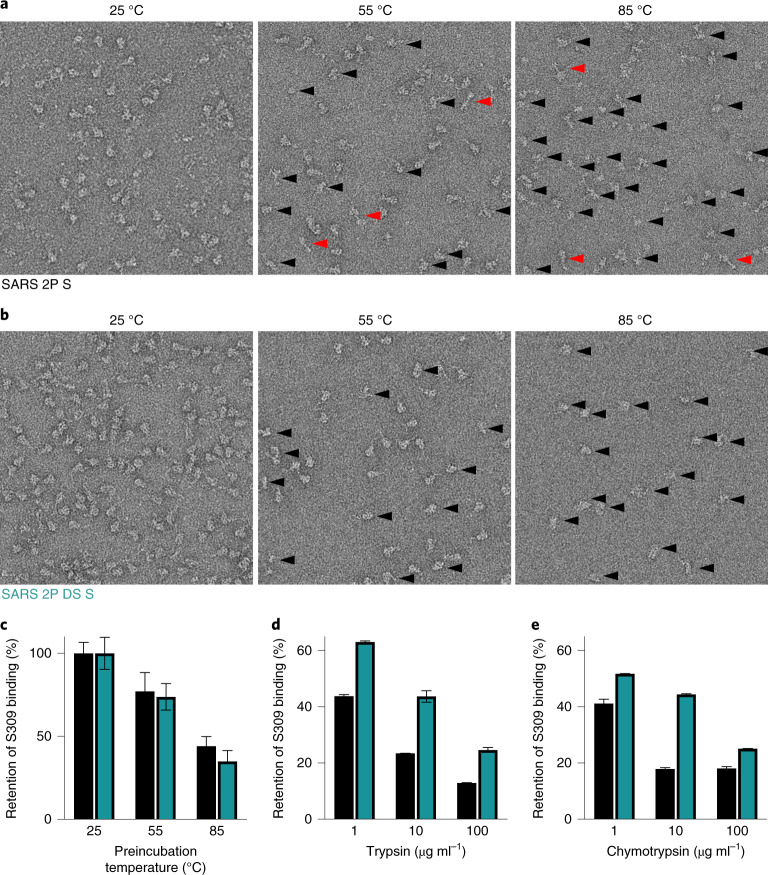
Evaluation of SARS-CoV-2 2P DS S thermal stability and protease resistance. **a**,**b**, Electron microscopy analysis of negatively stained SARS-CoV-2 2P S (**a**) and SARS-CoV-2 2P DS S (**b**) incubated for 20 min at 25, 55 and 85 °C. Black arrows highlight particles that appear to be misfolded. Red arrows highlight particles that appear to be in the postfusion conformation. **c**–**e**, Binding of human neutralizing antibody S309 to immobilized SARS-CoV-2 2P DS S (green) or SARS-CoV-2 2P S (black) preincubated for 20 min at 25, 55 and 85 °C (**c**), or for 16 h at 4 °C with 1, 10 or 100 µg ml^−1^ trypsin (**d**) or chymotrypsin (**e**). Graphs show the area under the curve of binding of serially diluted concentrations of the human neutralizing antibody S309; data are shown as mean and s.d. of *n* = 2 technical replicates, and are representative of one (**d** and **e**) or two (**c**) independent experiments. Data behind graphs are available in Supplementary Data 1.

To understand the impact of the introduced disulfide bond on S resistance to physical and chemical stress, we compared the architecture and antigenicity of SARS-CoV-2 2P DS S and SARS-CoV-2 2P S in various conditions. We first assessed the thermostability of the SARS-CoV-2 2P DS S and SARS-CoV-2 2P S using electron microscopy analysis of negatively stained samples incubated for 20 min at three temperatures. Both constructs were monodisperse and well folded at 25 °C (Fig. 4a,b). Although SARS-CoV-2 2P DS S and SARS-CoV-2 2P S exhibited approximately the same proportions of properly folded and unfolded protein after incubation at 55 °C, rod-shaped postfusion trimers were only observed for SARS-CoV-2 2P S (Fig. 4a,b). After incubation at 85 °C, an even larger number of particles corresponded to denatured protein (Fig. 4a,b). We subsequently used binding to the S309 monoclonal antibody to estimate retention of epitope integrity after preincubation at several temperatures. We observed decreased S309 binding as a function of temperature for both SARS-CoV-2 2P DS S and SARS-CoV-2 2P S, consistent with the electron microscopy data (Fig. 4c). Overall, these data indicate that both samples had comparable resistance to thermal denaturation but only SARS-CoV-2 2P DS S was entirely unable to transition from the prefusion to the postfusion state as a result of covalent stapling.

Based on our previous observation that SARS-CoV 2P S is sensitive to proteolysis by trypsin and chymotrypsin^69^, we set out to compare the proteolytic resistance of SARS-CoV-2 2P DS S and SARS-CoV-2 2P S side-by-side by ELISA. Using retention of S309 binding, we found that SARS-CoV-2 2P DS S was less sensitive than SARS-CoV-2 2P S to enzymatic digestion by either protease at a range of concentrations between 1 and 100 µg ml^−1^ (Fig. 4d,e). We speculate that the more rigid architecture of SARS-CoV-2 2P DS S correlates with its enhanced protease resistance compared with SARS-CoV-2 2P S, due to reduced conformational freedom.

### Disulfide stapling is broadly applicable to β-coronaviruses

**Fig. 5:**
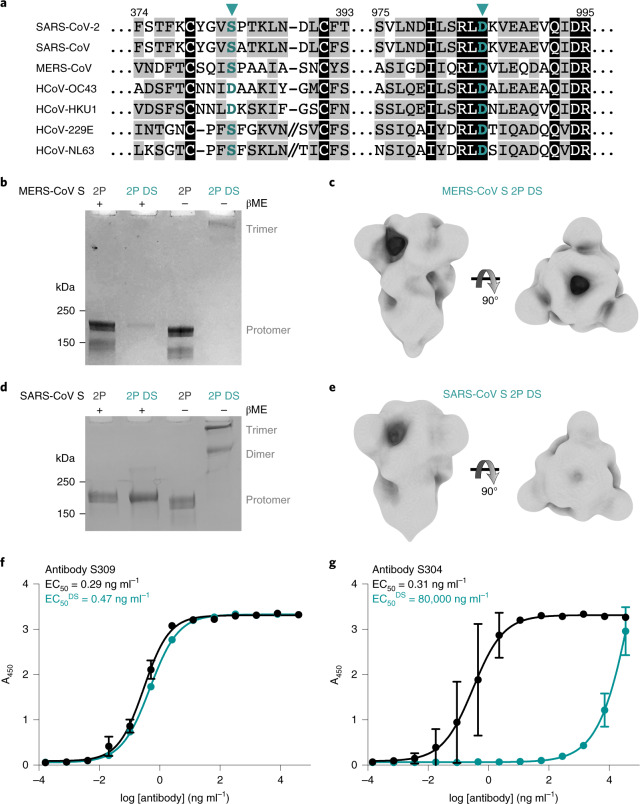
Design and validation of SARS-CoV 2P DS S and MERS-CoV 2P DS S. **a**, Sequence alignment showing the conservation of the residues involved in and adjacent to the designed disulfide bond across human coronavirus S glycoproteins. Residues are highlighted if they are identical in the alignment (black) or conservatively substituted (gray). Residues are numbered according to the SARS-CoV-2 S sequence. Green triangles highlight residues involved in the designed disulfide bond. **b**, SDS–PAGE analysis of MERS-CoV 2P S and MERS-CoV 2P DS S in reducing and nonreducing conditions showing formation of an intermolecular disulfide bond. **c**, 3D reconstruction in two orthogonal orientations of negatively stained MERS-CoV 2P DS S confirming proper folding of the designed protein construct. **d**, SDS–PAGE analysis of SARS-CoV 2P S and SARS-CoV 2P DS S in reducing and nonreducing conditions showing formation of an intermolecular disulfide bond. **e**, 3D reconstruction in two orthogonal orientations of negatively stained SARS-CoV 2P DS S confirming proper folding of the designed protein construct. **f**,**g**, Binding of various concentrations of the human neutralizing antibodies S309 (**f**) and S304 (**g**) to immobilized SARS-CoV 2P DS S (green) or SARS-CoV 2P S (black). Data are shown as mean and s.d. of *n* = 2 technical replicates; data are representative of two independent experiments.

Next, we set out to test the general applicability of the DS stabilizing strategy identified for SARS-CoV-2 S to other coronavirus S glycoproteins. Based on the high sequence and structural conservation of the residues involved in—and adjacent to—the engineered disulfide bond (Fig. 5a), we hypothesized that the DS mutation might be transferable to SARS-CoV S and MERS-CoV S, which share ~80% and ~30% sequence identity with SARS-CoV-2 S, respectively.

SDS–PAGE analysis of recombinantly expressed MERS-CoV 2P DS S (S429C/D1059C mutant) confirmed that it formed a high-molecular-weight species in nonreducing conditions, consistent with covalent formation of S homotrimers (Fig. 5b). Likewise, the electrophoretic mobility corresponded to individual S protomers in presence of β-mercaptoethanol. Electron microscopy analysis of negatively stained MERS-CoV 2P DS S demonstrated the engineered mutant folds as a globular closed homotrimer (Fig. 5c).

Similarly, SDS–PAGE analysis of recombinantly expressed SARS-CoV 2P DS S (S370C/D969C mutant) also confirmed that it formed a high-molecular-weight species in nonreducing conditions, consistent with covalent formation of S homotrimers (Fig. 5d). Homodimers were also observed for SARS-CoV 2P DS S. Electron microscopy analysis of negatively stained SARS-CoV 2P DS S demonstrated that it folds as a globular closed homotrimer (Fig. 5e).

Finally, we observed comparable dose-dependent binding of the S309 neutralizing antibody to SARS-CoV 2P DS S and SARS-CoV 2P S, validating retention of antigenicity of the designed construct (Fig. 5f). Similar to our findings with SARS-CoV-2 2P DS S, we observed a five-orders-of-magnitude-decreased binding of the S304 antibody to SARS-CoV 2P DS S compared with SARS-CoV 2P S (Fig. 5g), consistent with covalent S^B^ domain closure.

## Discussion

Viral glycoprotein engineering is an active field of research fueling vaccine design strategies to elicit potent and/or broad protection against a range of emerging or endemic pathogens. Stabilization of the respiratory syncytial virus fusion glycoprotein in its prefusion conformation (DS-Cav1)^51^ and subsequent fusion to a computationally designed trimeric protein (I53-50A)^70^ are recent breakthroughs illustrating the power of structure-based vaccine design. Ds-Cav1 is currently evaluated in a phase I randomized, open-label clinical trial to assess its safety, tolerability and immunogenicity in healthy adults (NCT03049488). Furthermore, multivalently displayed Ds-Cav1 genetically fused to the I53-50 nanoparticle has been shown to further improve elicitation of high titers of neutralizing antibodies^70^. Similarly, the development of HIV-1 SOSIP constructs has revolutionized the field of HIV-1 structural vaccinology and immunology^47^.

The recent emergence of SARS-CoV-2, the virus responsible for the ongoing COVID-19 pandemic, showcases the urgent need to explore strategies to expedite coronavirus vaccines and therapeutics design initiatives as well as structural and serology studies. Prefusion stabilization of MERS-CoV S through the aforementioned introduction of two proline substitutions was previously shown to elicit enhanced neutralizing antibody titers against multiple MERS-CoV isolates in mice^20^. However, the limited stability and conformational dynamics of the SARS-CoV-2 2P S, SARS-CoV 2P S and MERS-CoV 2P S ectodomain trimers indicate that further improvements are needed to increase their shelf life and/or to manipulate their conformational states.

We report here a strategy to produce prefusion-stabilized, closed coronavirus ectodomain trimers and show that it is broadly applicable to at least the most-pathogenic members; that is, SARS-CoV-2, SARS-CoV and MERS-CoV. By symmetrizing and stabilizing S proteins, we expect the DS mutation to be a useful tool for the research community, enabling high-resolution structural studies of antibody complexes, and for characterizing the humoral immune response in infected or vaccinated individuals and animals.

We hypothesize that the design strategy described here might improve the breadth of neutralizing antibodies elicited via masking of the highly immunogenic RBM, which is poorly conserved across distinct coronaviruses. The tradeoff, however, will be dampening of antibody titers targeting the RBM that is typically recognized by potent neutralizing antibodies but with narrow breadth between coronaviruses. For the same reasons, we also envisage that S glycoprotein constructs shut in the closed conformation could assist in isolating broadly neutralizing antibodies effective against multiple viruses belonging to distinct (sub)genera.

Finally, as demonstrated here, comparing the reactivity of DS constructs with protein constructs exhibiting the full range of S^B^ receptor-binding domain conformations will allow evaluation of the fraction of antibodies recognizing the RBM and/or cryptic epitopes in serology studies, to provide a detailed understanding of the humoral immune response elicited upon infection or vaccination. Given that RBM-targeting antibodies are typically neutralizing, this comparison may serve as a proxy for estimating whether or not a patient has neutralizing antibodies.

## Methods

### Design of disulfide mutants

Disulfide mutants were designed visually or using the Disulfide by Design 2 software^64^ and synthetic genes ordered from GenScript.

### Recombinant S ectodomains production

All ectodomains were produced in 500-ml cultures of HEK293F cells grown in suspension using FreeStyle 293 expression medium (Life technologies) at 37°C in a humidified 8% CO_2_ incubator rotating at 130 r.p.m., as previously reported^18^. The culture was transfected using 293fectin (ThermoFisher) with cells grown to a density of 10^6^ cells per ml and cultivated for 3 d. The supernatant was collected and cells were resuspended for another 3 d, yielding two collections. Clarified supernatants were purified using a 5-ml Cobalt affinity column (Takara). Purified protein was concentrated, and flash frozen in a buffer containing 50 mM Tris pH 8.0 and 150 mM NaCl before cryo-EM analysis.

### Antibody expression

Recombinant antibodies were expressed in ExpiCHO cells transiently cotransfected with plasmids expressing the heavy and light chains, as previously described^57^.

### Serum preparation

A de-identified serum sample from a patient with COVID-19 was collected and heat-inactivated at 56 °C for 1 h. The patient tested positive for SARS-CoV-2 5 d before the serum sample was taken. The sample collection and this study were approved by the Institutional Review Boards of the University of Washington. This study was granted a waiver of consent since it used residual clinical samples and existing clinical data.

### ELISA

First, 20 µl of ectodomains (stabilized prefusion trimer) of S from SARS-CoV-2 or SARS-CoV, or the disulfide-stabilized SARS-CoV-2 or SARS-CoV, were coated on 384-well ELISA plates at 1 ng µl^−1^ for 16 h at 4 °C. For thermal denaturation experiments, the S ectodomains were preincubated at 25, 55 or 85 °C for 20 min before coating the ELISA plates for 16 h at 4 °C. For protease sensitivity experiments, the S ectodomains were preincubated with 1, 10 or 100 µg ml^−1^ trypsin or chymotrypsin for 30 min at 25 °C before coating the ELISA plates, where the proteolysis reaction continued for 16 h at 4 °C. Plates were washed with a 405 TS Microplate Washer (BioTek Instruments) then blocked with 80 µl of SuperBlock (PBS) Blocking Buffer (Thermo Scientific) for 1 h at 37 °C. Plates were then washed and 30 µl of antibodies or human ACE2-Fc protein (Sino Biological) was added to the plates at concentrations between 0.001 and 100,000 ng ml^−1^ and incubated for 1 h at 37 °C. Plates were washed and then incubated with 30 µl of 1:5,000 diluted goat anti-human Fc IgG-HRP (Invitrogen, A18817). Plates were washed and then 30 µl of Substrate TMB Microwell Peroxidase (Seracare 5120-0083) was added for 5 min at room temperature. The colorimetric reaction was stopped by addition of 30 µl of 1 M HCl. A_450_ was read on a Varioskan Lux plate reader (Thermo Scientific) and plotted with a nonlinear regression curve fit using Prism 8.

### Pseudovirus neutralization assays

Murine leukemia virus (MLV)-based SARS-CoV-2 S-pseudotyped viruses were prepared as previously described^18^. HEK293T cells were cotransfected with a SARS-CoV-2 S encoding-plasmid, an MLV Gag-Pol packaging construct and the MLV transfer vector encoding a luciferase reporter using the Lipofectamine 2000 transfection reagent (Life Technologies) according to the manufacturer’s instructions. Cells were incubated for 5 h at 37 °C with 8% CO_2_ with OPTIMEM transfection medium. DMEM containing 10% FBS was added for 72 h.

BHK cells transiently transfected with human ACE2 were cultured in DMEM containing 10% FBS and 1% PenStrep, and plated into 96-well plates for 16–24 h. Concentrated pseudovirus with or without serial dilution of COVID-19 convalescent plasma was incubated for 1 h and then added to the wells after washing three times with DMEM. After 2–3 h, DMEM containing 20% FBS and 2% PenStrep was added to the cells for 48 h. Following 48 h of infection, One-Glo-EX (Promega) was added to the cells and incubated in the dark for 5–10 min before reading on a Varioskan LUX plate reader (ThermoFisher). Measurements were done in duplicate, and relative luciferase units were converted to percentage neutralization and plotted with a nonlinear regression curve fit in Prism 8.

### Negative-stain EM sample preparation

All constructs in this study were negatively stained at a final concentration of 0.06 mg ml^−1^ using Gilder Grids overlaid with a thin layer of carbon and 2% uranyl formate. For thermal denaturation experiments, 0.06 mg ml^−1^ S ectodomains were preincubated at 25, 55 or 85 °C for 20 min before being added to grids. Data were acquired using the Leginon software^71^ to control a Tecnai T12 transmission electron microscope operated at 120 kV and equipped with a Gatan 4K Ultrascan CCD detector. The dose rate was adjusted to 50 electrons per Å^2^ and each micrograph was acquired in 1 s. In a single session, ~100 micrographs were collected with a defocus range between −1.0 and −2.5 μm. Data were subsequently processed using cryoSPARC^72^.

### Cryo-EM sample preparation and data collection

First, 3 µl of SARS-CoV-2 2P DS S at 0.5 mg ml^−1^ was applied onto a freshly glow-discharged 2.0/2.0 UltraFoil grid (200 mesh). Plunge freezing used a Vitrobot Mark IV (ThermoFisher) using a blot force of 0 and 6.5-s blot time, at 100% humidity and 23 °C. Data were acquired using the Leginon software^71^ to control a Glacios transmission electron microscope operated at 200 kV and equipped with a Gatan K2 Summit direct detector. The dose rate was adjusted to 8 counts per pixel per second, and each movie was acquired in 50 frames of 200 ms with a pixel size of 1.16 Å at the specimen level. In a single session, ~600 micrographs were collected with a defocus range between −0.8 and −3.0 μm.

### Cryo-EM data processing

Movie frame alignment, estimation of the microscope contrast transfer function parameters, particle picking and extraction (with a box size of 352 pixels^2^) were carried out using Warp^73^. Reference-free two-dimensional classification was performed using cryoSPARC^72^ to select well-defined particle images. 3D classifications with 50 iterations each (angular sampling 7.5° for 25 iterations and 1.8° with local search for 25 iterations) were carried out using Relion^74^ without imposing symmetry to separate distinct SARS-CoV-2 S conformations. 3D refinements were carried out using nonuniform refinement along with per-particle defocus refinement in cryoSPARC^72^. Particle images were subjected to Bayesian polishing^75^ before performing another round of nonuniform refinement in cryoSPARC^72^ followed by per-particle defocus refinement and, again, nonuniform refinement. Reported resolutions are based on the gold-standard Fourier shell correlation (FSC) of 0.143 criterion and FSC curves were corrected for the effects of soft masking by high-resolution noise substitution^76^.

### Cryo-EM model building and analysis

UCSF Chimera^77^ and Coot were used to fit an atomic model (PDB 6VXX) into the cryo-EM map. The model was then refined into the map using Rosetta^78–80^ and analyzed using MolProbity^81^, EMringer^82^ and Phenix^83^. Figures were generated using UCSF ChimeraX^84^ and UCSF Chimera^77^.

### Reporting Summary

Further information on research design is available in the Nature Research Reporting Summary linked to this article.

## Online content

Any methods, additional references, Nature Research reporting summaries, source data, extended data, supplementary information, acknowledgements, peer review information; details of author contributions and competing interests; and statements of data and code availability are available at 10.1038/s41594-020-0483-8.

## Supplementary information

Reporting Summary

Peer Review Information

Supplementary Data 1 Uncropped images for Figs. 1d and 5b,d and statistical source data behind graphs for Figs. 3a–f, 4c–e and 5f,g.

## Data Availability

The cryo-EM map and atomic model have been deposited to the EMDB and wwPDB with accession codes EMD-22083 and PDB 6X79, respectively.

## Extended data

**Extended data** is available for this paper at 10.1038/s41594-020-0483-8.
