# Supplementary material for: Structure-guided covalent stabilization of coronavirus spike glycoprotein trimers in the closed conformation
Source: Nat Struct Mol Biol. 2020 Aug 4;27(10):942–9. doi: 10.1038/s41594-020-0483-8 (PMC7541350; doi:10.1038/s41594-020-0483-8)
Supplement: Supplementary file 1 — Reporting Summary [file 41594_2020_483_MOESM1_ESM.pdf]

nature research

Corresponding author(s): *Double-blind peer review submissions: write DBPR and your manuscript number here instead of author names.*

Last updated by author(s): *YYYY-MM-DD*

Reporting Summary

Nature Research wishes to improve the reproducibility of the work that we publish. This form provides structure for consistency and transparency in reporting. For further information on Nature Research policies, see our Editorial Policies and the Editorial Policy Checklist.

Statistics

For all statistical analyses, confirm that the following items are present in the figure legend, table legend, main text, or Methods section.

n/a	Confirmed	
☐	☒	The exact sample size (n) for each experimental group/condition, given as a discrete number and unit of measurement
☐	☒	A statement on whether measurements were taken from distinct samples or whether the same sample was measured repeatedly
☐	☒	The statistical test(s) used AND whether they are one- or two-sided<br>*Only common tests should be described solely by name; describe more complex techniques in the Methods section.*
☐	☒	A description of all covariates tested
☐	☒	A description of any assumptions or corrections, such as tests of normality and adjustment for multiple comparisons
☐	☒	A full description of the statistical parameters including central tendency (e.g. means) or other basic estimates (e.g. regression coefficient) AND variation (e.g. standard deviation) or associated estimates of uncertainty (e.g. confidence intervals)
☒	☐	For null hypothesis testing, the test statistic (e.g. F, t, r) with confidence intervals, effect sizes, degrees of freedom and P value noted<br>*Give P values as exact values whenever suitable.*
☒	☐	For Bayesian analysis, information on the choice of priors and Markov chain Monte Carlo settings
☒	☐	For hierarchical and complex designs, identification of the appropriate level for tests and full reporting of outcomes
☒	☐	Estimates of effect sizes (e.g. Cohen's d, Pearson's r), indicating how they were calculated

Our web collection on statistics for biologists contains articles on many of the points above.

Software and code

Policy information about availability of computer code

Data collection	Electron microscopy: Leginon software<br>ELISA: Varioskan Lux plate reader with SkanIt RE 5.0
Data analysis	Electron microscopy: cryoSPARC v2, warp, relion 3.0, UCSF Chimera, Coot, Rosetta, Phenix, MolProbity, and EMringer<br>ELISA: Prism 8

For manuscripts utilizing custom algorithms or software that are central to the research but not yet described in published literature, software must be made available to editors and reviewers. We strongly encourage code deposition in a community repository (e.g. GitHub). See the Nature Research guidelines for submitting code & software for further information.

Data

Policy information about availability of data

All manuscripts must include a data availability statement. This statement should provide the following information, where applicable:

- Accession codes, unique identifiers, or web links for publicly available datasets
- A list of figures that have associated raw data
- A description of any restrictions on data availability

Figure 2: The cryo-EM map and atomic model have been deposited to the EMDB and wwPDB with accession numbers EMD-22083 and PDB 6X79, respectively.
Figures 1, 3, 4, and 5: SDS-PAGE gels and raw data are available in the Source Data

Field-specific reporting

Please select the one below that is the best fit for your research. If you are not sure, read the appropriate sections before making your selection.

☒ Life sciences ☐ Behavioural & social sciences ☐ Ecological, evolutionary & environmental sciences

For a reference copy of the document with all sections, see nature.com/documents/nr-reporting-summary-flat.pdf

Life sciences study design

All studies must disclose on these points even when the disclosure is negative.

Sample size	n=2 was chosen for the ELISAs as the variability is typically quite low
Data exclusions	N/A
Replication	Where possible, data was reproduced at least twice to verify its reproducibility.
Randomization	N/A
Blinding	N/A

Reporting for specific materials, systems and methods

We require information from authors about some types of materials, experimental systems and methods used in many studies. Here, indicate whether each material, system or method listed is relevant to your study. If you are not sure if a list item applies to your research, read the appropriate section before selecting a response.

Materials & experimental systems

n/a	Involved in the study	
☐	☒	Antibodies
☐	☒	Eukaryotic cell lines
☒	☐	Palaeontology and archaeology
☒	☐	Animals and other organisms
☐	☒	Human research participants
☒	☐	Clinical data
☒	☐	Dual use research of concern

Methods

n/a	Involved in the study	
☒	☐	ChIP-seq
☒	☐	Flow cytometry
☒	☐	MRI-based neuroimaging

Antibodies

Antibodies used	Recombinant antibodies were expressed in ExpiCHO cells transiently co-transfected with plasmids expressing the heavy and light chain. The sequences for S309 and S304 were originally obtained from SARS-CoV patients, and S2H14 was obtained from a SARS-CoV-2 patient.
Validation	Binding of these antibodies has been validated elsewhere.

Eukaryotic cell lines

Policy information about cell lines

Cell line source(s)	HEK 293F and HEK 293T were obtained from Thermofisher.
Authentication	None of the cell lines used were authenticated.
Mycoplasma contamination	The cell lines were not tested for mycoplasma contamination.
Commonly misidentified lines (See ICLAC register)	N/A

Human research participants

Policy information about studies involving human research participants

Population characteristics	N/A
Recruitment	A de-identified COVID-19 patient serum sample was collected after the patient tested positive for SARS-CoV-2 five days earlier.
Ethics oversight	The sample collection and this study were approved by the Institutional Review Boards of the University of Washington. This study was granted a waiver of consent since it used residual clinical samples and existing clinical data.

Note that full information on the approval of the study protocol must also be provided in the manuscript.
