# Supplementary material for: Structure-guided covalent stabilization of coronavirus spike glycoprotein trimers in the closed conformation
Source: Nat Struct Mol Biol. 2020 Aug 4;27(10):942–9. doi: 10.1038/s41594-020-0483-8 (PMC7541350; doi:10.1038/s41594-020-0483-8)
Supplement: Supplementary file 2 — Peer Review Information [file 41594_2020_483_MOESM2_ESM.pdf]

# Peer Review Information

**Journal:** Nature Structural and Molecular Biology
**Manuscript Title:** Structure-guided covalent stabilization of coronavirus spike glycoprotein trimers in the closed conformation
**Corresponding author name(s):** Prof. David Veesler

## Reviewer Comments & Decisions:

**Decision Letter, initial version:**

17th Jun 2020

Dear David,

Thank you again for submitting your manuscript "Closing coronavirus spike glycoproteins by structure-guided design". We now have comments (below) from the 2 reviewers who evaluated your paper. In light of those reports, we remain interested in your study and would like to see your response to the comments of the referees, in the form of a revised manuscript.

You will see that both reviewers are positive about the interest and timeliness of the work. Reviewer 1 requests directly measuring and comparing the thermostability of the constructs; both reviewers ask about expression level and yield of the constructs. They also have suggestions to improve presentation and clarity, with changes to text and figures.

Please be sure to address/respond to all concerns of the referees in full in a point-by-point response and highlight all changes in the revised manuscript text file. If you have comments that are intended for editors only, please include those in a separate cover letter.

In addition, the manuscript requires changes to conform to our editorial style and requirements. I have marked those changes in the attached document. Of note:
- in the methods section (and reporting summary), please make sure to include information about the IRB committee and a statement confirming that informed consent was obtained.
- make sure to define all error bars; add error bars to curve shown in Suppl Fig 3.
- all supplementary figures should be relabeled as "Extended Data Figures" (limit is 10).

We are committed to providing a fair and constructive peer-review process. Do not hesitate to contact us if there are specific requests from the reviewers that you believe are technically impossible or

unlikely to yield a meaningful outcome.

We expect to see your revised manuscript within 2 weeks. If you cannot send it within this time, please contact us to discuss an extension; we would still consider your revision, provided that no similar work has been accepted for publication at NSMB or published elsewhere.

As you already know, we put great emphasis on ensuring that the methods and statistics reported in our papers are correct and accurate. As such, if there are any changes that should be reported, please submit an updated version of the Reporting Summary along with your revision.

Please follow the links below to download these files:

Reporting Summary:
https://www.nature.com/documents/nr-reporting-summary.pdf

Please note that the form is a dynamic 'smart pdf' and must therefore be downloaded and completed in Adobe Reader.

If there are additional or modified structures presented in the final revision, please submit the corresponding PDB validation reports.

SOURCE DATA: we urge authors to provide, in tabular form, the data underlying the graphical representations used in figures. This is to further increase transparency in data reporting, as detailed in this editorial (http://www.nature.com/nsmb/journal/v22/n10/full/nsmb.3110.html). Spreadsheets can be submitted in excel format. Only one (1) file per figure is permitted; thus, for multi-paneled figures, the source data for each panel should be clearly labeled in the Excel file; alternatively, the data can be provided as multiple, clearly labeled sheets in a single Excel file. When submitting files, the title field should indicate which figure the source data pertains to. We encourage our authors to provide source data at the revision stage, so that they are part of the peer-review process.

Data availability: this journal strongly supports public availability of data. All data used in accepted papers should be available via a public data repository, or alternatively, as Supplementary Information. If data can only be shared on request, please explain why in your Data Availability Statement, and also in the correspondence with your editor. Please note that for some data types, deposition in a public repository is mandatory - more information on our data deposition policies and available repositories can be found below:
https://www.nature.com/nature-research/editorial-policies/reporting-standards#availability-of-data

We require deposition of coordinates (and, in the case of crystal structures, structure factors) into the Protein Data Bank with the designation of immediate release upon publication (HPUB). Electron microscopy-derived density maps and coordinate data must be deposited in EMDB and released upon publication. Deposition and immediate release of NMR chemical shift assignments are highly encouraged. Deposition of deep sequencing and microarray data is mandatory, and the datasets must be released prior to or upon publication. To avoid delays in publication, dataset accession numbers must be supplied with the final accepted manuscript and appropriate release dates must be indicated at the galley proof stage.

While we encourage the use of color in preparing figures, please note that this will incur a charge to partially defray the cost of printing. Information about color charges can be found at http://www.nature.com/nsmb/authors/submit/index.html#costs

Nature Structural & Molecular Biology is committed to improving transparency in authorship. As part of our efforts in this direction, we are now requesting that all authors identified as 'corresponding author' on published papers create and link their Open Researcher and Contributor Identifier (ORCID) with their account on the Manuscript Tracking System (MTS), prior to acceptance. This applies to primary research papers only. ORCID helps the scientific community achieve unambiguous attribution of all scholarly contributions. You can create and link your ORCID from the home page of the MTS by clicking on 'Modify my Springer Nature account'. For more information please visit please visit <a href="http://www.springernature.com/orcid">www.springernature.com/orcid</a>.

Please use the link below to submit your revised manuscript and related files:

[REDACTED]

<strong>Note:</strong> This URL links to your confidential home page and associated information about manuscripts you may have submitted, or that you are reviewing for us. If you wish to forward this email to co-authors, please delete the link to your homepage.

We look forward to seeing the revised manuscript and thank you for the opportunity to review your work.

Sincerely,

Ines Chen, Ph.D.
Chief Editor
Nature Structural & Molecular Biology

ORCID 0000-0002-1405-9703

Referee expertise:

Referee #1: structural, virus-antibody

Referee #2: coronavirus

Reviewers' Comments:

Reviewer #1:
Remarks to the Author:
McCallum et al. report design of coronavirus S protein trimers in the closed, prefusion conformation by using disulfide bonds to lock the RBDs in the 'down' conformation. The new DS constructs for both

SARS-CoV-2 and SARS-CoV S trimers will be useful tools for evaluating antibodies from patient plasmas and is suitable for publication in NSMB with some changes (and hopefully experiments to evaluate stabilities and make more DS constructs). The major problem with the writing is that the authors don't review the literature about 'up' and 'down' RBD conformations and how these relate to receptor binding and antibody recognition in the introduction, so the reader has to already be familiar with the field to interpret the motivation for the study and its importance.

1) The title of the paper, "Closing the coronavirus spike glycoproteins by structure-guided design," sounds a bit odd since some readers will not know what "closing" means in this context. Maybe something like "Covalent stabilization of prefusion conformation coronavirus spike glycoproteins" or a variant of that would be a better title.

2) A summary of S trimer structures that include 'up' and 'down' SB (RBD) domains in the introduction, including how this relates to ACE2 receptor binding, would be helpful.

3) The criteria for choosing residues for the three inter-molecular disulfide bonds (S383C/D985C, G413C/P987C, and T385C/T415C) should be discussed.

4) How do expression levels differ for the DS versions of the S trimers?

5) Details of the patient plasma sample (e.g., how long after infection was the sample collected) should be provided.

6) The authors speculate that the engineered SARS-CoV-2 2P DS S construct has enhanced stability relative to the construct lacking the DS mutations based on solving a (very nice) 2.9 Å single-particle cryo-EM structure of the closed S trimer using many fewer particles than other studies. The number of particles used in the other studies should be listed along with their resolutions, and the authors should discuss whether the other studies applied 3-fold symmetry (presumably only for the portion of trimers with all three SB domains in the closed conformation). Also, the authors should do some sort of thermostability assay to experimentally evaluate stability of the engineered construct compared with the original construct.

7) I think I understand what is mean in the following sentence, but it should be revised: "The high structural similarity between the SARS-CoV-2 2P DS S structure presented here and our previously reported SARS-CoV-2 2P S structure (in the closed conformation)18, led us to hypothesize that they would have similar antigenicity profiles – besides the lost ability to interact with binders requiring opening of the SB receptor-binding domains (e.g. the receptor-binding motif) for the former construct." For example, it should be noted that the engineered closed construct cannot bind to ACE2.

8) This sentence in the Discussion is somewhat misleading: "We report here a strategy to produce prefusion-stabilized, closed coronavirus ectodomain trimers and show it is broadly applicable to at least the β-genus which accounts for 5 out of 7 human-infecting coronaviruses, including the most pathogenic members (i.e. SARS-CoV-2, SARS-CoV and MERS-CoV)." The strategy cannot be said to be "broadly applicable" when it was only applied to S proteins from SARS-CoV-2 and SARS-CoV. The paper would be stronger if the authors reported results of introducing this mutation into other b-coronaviruses S trimers.

9) This word "comprised" in this sentence from the Methods makes no sense: "~600 micrographs

were collected in a single session with a defocus range comprised between -0.8 and -3.0 µm."

10) Supplemental Fig. 3 should include error bars.

11) A density map for the region surrounding the introduced disulfide bond (not just the disulfide density in Fig. 2d) should be shown in a figure.

Reviewer #2:
Remarks to the Author:
This is a valuable report on protein engineering. Using knowledge of SARS-CoV-2 spike protein structure, the authors introduced disulfide bonds, expecting that the bonds would tether a flexible domain into a stable closed conformation. This strategy in which engineered disulfides stabilize protein conformations is not original, but applied here to SARS-CoV-2, the positive results from this molecular stapling are very highly significant.

The submission scores very high for validating the molecular stapling approach with coronavirus spikes. The paper includes high quality structural results documenting disulfide (DS) closure of the flexible coronavirus spike domains. The report promotes the closed DS mutants as tools for antibody discovery, antibody epitope analysis, and vaccinology. The study documents the utility of the closed spikes in distinguishing RBD from non-RBD specific antibodies. Given the current goals to characterize humoral responses to SARS-CoV-2, and to evaluate COVID19 vaccines, the report is relevant and timely.

The presentation and the clarity of the text are excellent, and conclusions are supported. There are only a few comments on textual matters and figures.

Overall, the report makes a significant advance in understanding coronavirus spike protein structure and also provides valuable new reagents for the field. There are no functional analyses of the DS mutants, i.e., the DS mutations were not introduced into complete spikes to assess impacts on native spike functions in cell binding and virus entry, but in this reviewer's opinion, such functional analyses are beyond the scope of the study.

Some comments:
1. In the text about S1 subunit shedding, it appears as though authors are saying shedding has been observed only for SARS-CoV-2, SARS-CoV, and MERS-CoV. If yes, then this would not be the case. Shedding was first observed with MHV coronavirus and SARS-CoV S1 does not shed.
2. Were the S383C / D985C yields similar to those without these two mutations? (similar folding and secretion efficiencies)?
3. Fig 1c does show the locations of the DS links but only in a close-up view and Fig 2d shows even more close-up. Consider designating the location of the DS link in Fig 2b or 2c so that the readers can get a better sense of their locations.
4. The text referring to the closed spike structures being able to "complement tests using ACE2 inhibition as a proxy for evaluating the presence of neutralizing antibody titers" is confusing. Is it that the closed spikes, in parallel with open spikes, can distinguish neutralizing S domain B specific antibodies from other spike antibodies? Whether yes or no, clarifying this sentence may be helpful.

## Author Rebuttal to Initial comments

We would like to thank the reviewers for their constructive feedback which allowed us to improve the revised version of this manuscript.

**Reviewers' Comments:**

**Reviewer #1:** McCallum et al. report design of coronavirus S protein trimers in the closed, prefusion conformation by using disulfide bonds to lock the RBDs in the 'down' conformation. The new DS constructs for both SARS-CoV-2 and SARS-CoV S trimers will be useful tools for evaluating antibodies from patient plasmas and is suitable for publication in NSMB with some changes (and hopefully experiments to evaluate stabilities and make more DS constructs). The major problem with the writing is that the authors don't review the literature about 'up' and 'down' RBD conformations and how these relate to receptor binding and antibody recognition in the introduction, so the reader has to already be familiar with the field to interpret the motivation for the study and its importance.
**Author Response:** Thank you very much for seeing the utility of this study. Most of this information was already introduced at the beginning of the Results. Following the reviewer's suggestion, we moved this section to the Introduction and further elaborated on open and closed $S^B$ conformations, as follows:

"Structural fluctuations of the receptor-binding $S^B$ domain (also known as RBD), from a closed to an open conformation, enables exposure of the receptor-binding motif (RBM) which mediates interaction with angiotensin-converting enzyme 2 (ACE2) for SARS-CoV-2[6,18,26-31] and SARS-CoV[32,33], or dipeptidyl-peptidase 4 for MERS-CoV[34,35] **(Fig 1 a-b)**."

**Reviewer #1:** 1) The title of the paper, "Closing the coronavirus spike glycoproteins by structure-guided design," sounds a bit odd since some readers will not know what "closing" means in this context. Maybe something like "Covalent stabilization of prefusion conformation coronavirus spike glycoproteins" or a variant of that would be a better title.

**Author Response:** Excellent suggestion, we have adjusted the title to be "Structure-guided covalent stabilization of coronavirus spike glycoprotein trimers in the closed conformation".

**Reviewer #1:** 2) A summary of S trimer structures that include 'up' and 'down' SB (RBD) domains in the introduction, including how this relates to ACE2 receptor binding, would be helpful.
**Author Response:** This has been updated accordingly, see above for details.

**Reviewer #1:** 3) The criteria for choosing residues for the three inter-molecular disulfide bonds (S383C/D985C, G413C/P987C, and T385C/T415C) should be discussed.
**Author Response:** S383C/D985C was chosen because it was identified by Disulphide by design web-server. G413C/P987C and T385C/T415C were chosen because their carbon-alpha atoms were

approximately 6 Å apart. We've included the text, "S383C/D985C was identified by Disulphide by Design web-server, while G413C/P987C and T385C/T415C were selected manually."

**Reviewer #1:** 4) How do expression levels differ for the DS versions of the S trimers?
**Author Response:** Expression is approximately 10 times less than the 2P S trimer. This has been included in the text, "The yield was about 10-fold reduced compared with SARS-CoV-2 2P S."

**Reviewer #1:** 5) Details of the patient plasma sample (e.g., how long after infection was the sample collected) should be provided.
**Author Response:** Additional details about the patient serum sample have been included as follows:

"A de-identified COVID-19 patient serum sample was collected and heat-inactivated at 56°C for one hour. The patient tested positive for SARS-CoV-2 five days before the serum sample was taken. The sample collection and this study were approved by the Institutional Review Boards of the University of Washington. This study was granted a waiver of consent since it used residual clinical samples and existing clinical data"

**Reviewer #1:** 6) The authors speculate that the engineered SARS-CoV-2 2P DS S construct has enhanced stability relative to the construct lacking the DS mutations based on solving a (very nice) 2.9 Å single-particle cryo-EM structure of the closed S trimer using many fewer particles than other studies. The number of particles used in the other studies should be listed along with their resolutions, and the authors should discuss whether the other studies applied 3-fold symmetry (presumably only for the portion of trimers with all three SB domains in the closed conformation). Also, the authors should do some sort of thermostability assay to experimentally evaluate stability of the engineered construct compared with the original construct.
**Author Response:** Based on the outcomes of the thermostability/limited proteolysis assays introduced in the revised version of the manuscript, we removed this section from the manuscript as other, cryo-EM-related factors could have contributed to achieving this resolution with so few particles (e.g. using gold grids, orientation distributions, particle homogeneity…).

**Reviewer #1:** 7) I think I understand what is mean in the following sentence, but it should be revised: "The high structural similarity between the SARS-CoV-2 2P DS S structure presented here and our previously reported SARS-CoV-2 2P S structure (in the closed conformation)18, led us to hypothesize that they would have similar antigenicity profiles – besides the lost ability to interact with binders requiring opening of the SB receptor-binding domains (e.g. the receptor-binding motif) for the former construct." For example, it should be noted that the engineered closed construct cannot bind to ACE2.
**Author Response:** We evaluated ACE2 binding in the revised version of the manuscript and showed that SARS-CoV-2 2P DS S bound ACE2 100x more weakly than SARS-CoV-2 2P S as expected (Fig3b) and in agreement with binding of the RBM-targeted S2H14 antibody (Fig 3c). We rephrased this section as follows:

“Given that the RBM is concealed in the closed conformation, we hypothesized that SARS-CoV-2 2P DS S binding to ACE2 and RBM-targeted antibodies would be hindered. As expected, ELISA experiments showed that SARS-CoV-2 2P DS S recognized ACE2 with two-orders of magnitude reduced binding response compared to SARS-CoV-2 2P S **(Fig. 3 b)**. Furthermore, the binding response of the RBM-targeted S2H14 antibody to SARS-CoV-2 2P DS S was also dampened by two orders of magnitude relative to SARS-CoV-2 2P S, due to conformational masking of the RBM in the closed conformation[18] **(Fig. 3 c)**.”

**Reviewer #1:** 8) This sentence in the Discussion is somewhat misleading: “We report here a strategy to produce prefusion-stabilized, closed coronavirus ectodomain trimers and show it is broadly applicable to at least the β-genus which accounts for 5 out of 7 human-infecting coronaviruses, including the most pathogenic members (i.e. SARS-CoV-2, SARS-CoV and MERS-CoV).” The strategy cannot be said to be “broadly applicable” when it was only applied to S proteins from SARS-CoV-2 and SARS-CoV. The paper would be stronger if the authors reported results of introducing this mutation into other b-coronaviruses S trimers.
**Author Response:** Thank you for this suggestion. We have tested expression for the DS mutant in the ectodomain of MERS S 2P. We observe trimers in a non-reducing SDS-PAGE gel and structurally sound trimers by negative stain. Thus we think that this strategy can be thought of as broadly applicable. These new results have been introduced in the text and Fig 5b-c.

**Reviewer #1:** 9) This word “comprised” in this sentence from the Methods makes no sense: “~600 micrographs were collected in a single session with a defocus range comprised between -0.8 and -3.0 μm.”
**Author Response:** The recommended edit has been made.

**Reviewer #1:** 10) Supplemental Fig. 3 should include error bars.
**Author Response:** The recommended edit has been made.

**Reviewer #1:** 11) A density map for the region surrounding the introduced disulfide bond (not just the disulfide density in Fig. 2d) should be shown in a figure.
**Author Response:** The recommended edit has been introduced as Extended Data Fig3.

Reviewer #2:
**Reviewer #2:** This is a valuable report on protein engineering. Using knowledge of SARS-CoV-2 spike protein structure, the authors introduced disulfide bonds, expecting that the bonds would tether a flexible domain into a stable closed conformation. This strategy in which engineered disulfides stabilize protein conformations is not original, but applied here to SARS-CoV-2, the positive results from this molecular stapling are very highly significant.
The submission scores very high for validating the molecular stapling approach with coronavirus spikes. The paper includes high quality structural results documenting disulfide (DS) closure of the flexible coronavirus spike domains. The report promotes the closed DS mutants as tools for antibody discovery, antibody epitope analysis, and vaccinology. The study documents the utility of the closed spikes in

distinguishing RBD from non-RBD specific antibodies. Given the current goals to characterize humoral responses to SARS-CoV-2, and to evaluate COVID19 vaccines, the report is relevant and timely.
The presentation and the clarity of the text are excellent, and conclusions are supported. There are only a few comments on textual matters and figures.
Overall, the report makes a significant advance in understanding coronavirus spike protein structure and also provides valuable new reagents for the field. There are no functional analyses of the DS mutants, i.e., the DS mutations were not introduced into complete spikes to assess impacts on native spike functions in cell binding and virus entry, but in this reviewer's opinion, such functional analyses are beyond the scope of the study.

**Author Response:** Thank you very much.

Some comments:
**Reviewer #2:** 1. In the text about S1 subunit shedding, it appears as though authors are saying shedding has been observed only for SARS-CoV-2, SARS-CoV, and MERS-CoV. If yes, then this would not be the case. Shedding was first observed with MHV coronavirus and SARS-CoV S1 does not shed.
**Author Response:** We apologize for the lack of clarity. We meant that only these three spike glycoproteins have been observed with open RBDs via structural studies. The text has been modified accordingly.

**Reviewer #2:** 2. Were the S383C / D985C yields similar to those without these two mutations? (similar folding and secretion efficiencies)?

**Author Response:** Yield after purification is about 10 fold less than without these two mutations, see response to reviewer #1 above.

**Reviewer #2:** 3. Fig 1c does show the locations of the DS links but only in a close-up view and Fig 2d shows even more close-up. Consider designating the location of the DS link in Fig 2b or 2c so that the readers can get a better sense of their locations.
**Author Response:** These figures have been adjusted to show the DS link and location better (revised Fig2c).

**Reviewer #2:** 4. The text referring to the closed spike structures being able to "complement tests using ACE2 inhibition as a proxy for evaluating the presence of neutralizing antibody titers" is confusing. Is it that the closed spikes, in parallel with open spikes, can distinguish neutralizing S domain B specific antibodies from other spike antibodies? Whether yes or no, clarifying this sentence may be helpful.

**Author Response:** Yes, this is what we meant. We apologize for the confusion. In the revised version of the manuscript, we provide new data to show that ACE2 binds two-logs less than without the DS mutation. Since antibodies that bind to the ACE2 binding site tend to be neutralizing antibodies and that this has been proposed to be used as a proxy for the presence of neutralizing antibodies, we suggest that comparing SARS-CoV-2 2P DS S and SARS-CoV-2 2P S will allow similar analyzes.

**Decision Letter, first revision:**

12th Jul 2020

Dear David,

Thank you again for submitting your manuscript "Structure-guided covalent stabilization of coronavirus spike glycoprotein trimers in the closed conformation". The comments from one of the original referees are below, and based on these comments, we are happy to accept your paper, in principle, for publication as an Article in Nature Structural & Molecular Biology, on the condition that you revise your manuscript in response to our editorial requirements.

The text and figures require revisions. Note that we will send you detailed instructions for the final revision, along with information on editorial and formatting requirements.

Data availability: this journal strongly supports public availability of data. Please place the data used in your paper into a public data repository, or alternatively, present the data as Supplementary Information. If data can only be shared on request, please explain why in your Data Availability Statement, and also in the correspondence with your editor. Please note that for some data types, deposition in a public repository is mandatory - more information on our data deposition policies and available repositories can be found below:
https://www.nature.com/nature-research/editorial-policies/reporting-standards#availability-of-data

TRANSPARENT PEER REVIEW
Nature Structural & Molecular Biology offers a transparent peer review option for new original research manuscripts submitted from 1st December 2019. We encourage increased transparency in peer review by publishing the reviewer comments, author rebuttal letters and editorial decision letters if the authors agree. Such peer review material is made available as a supplementary peer review file. <b>Please state in the cover letter 'I wish to participate in transparent peer review' if you want to opt in, or 'I do not wish to participate in transparent peer review' if you don't.</b> Failure to state your preference will result in delays in accepting your manuscript for publication.
Please note: we allow redactions to authors' rebuttal and reviewer comments in the interest of confidentiality. If you are concerned about the release of confidential data, please let us know specifically what information you would like to have removed. Please note that we cannot incorporate redactions for any other reasons. Reviewer names will be published in the peer review files if the reviewer signed the comments to authors, or if reviewers explicitly agree to release their name. For more information, please refer to our <a href="https://www.nature.com/documents/nr-transparent-peer-review.pdf" target="new">FAQ page</a>.

<b>ORCID</b>

Nature Structural & Molecular Biology is committed to improving transparency in authorship. As part of our efforts in this direction, we are now requesting that all authors identified as 'corresponding author' create and link their Open Researcher and Contributor Identifier (ORCID) with their account on

the Manuscript Tracking System (MTS) prior to acceptance. ORCID helps the scientific community achieve unambiguous attribution of all scholarly contributions. For more information please visit http://www.springernature.com/orcid

For all corresponding authors listed on the manuscript, please follow the instructions in the link below to link your ORCID to your account on our MTS before submitting the final version of the manuscript. If you do not yet have an ORCID you will be able to create one in minutes.
https://www.springernature.com/gp/researchers/orcid/orcid-for-nature-research

IMPORTANT: All authors identified as 'corresponding author' on the manuscript must follow these instructions. Non-corresponding authors do not have to link their ORCIDs but are encouraged to do so. Please note that it will not be possible to add/modify ORCIDs at proof. Thus, if they wish to have their ORCID added to the paper they must also follow the above procedure prior to acceptance.

To support ORCID's aims, we only allow a single ORCID identifier to be attached to one account. If you have any issues attaching an ORCID identifier to your MTS account, please contact the <a href="http://platformsupport.nature.com/">Platform Support Helpdesk</a>.

We hope that you will support this initiative and supply the required information. Should you have any query or comments, please do not hesitate to contact me.

If you have any questions, please do not hesitate to contact me directly.
Sincerely,

Ines Chen, Ph.D.
Chief Editor
Nature Structural & Molecular Biology

ORCID 0000-0002-1405-9703

Reviewer #1 (Remarks to the Author):

The authors have responded to suggestions from reviewers, and I think the article would now be suitable for publication in NSMB. The utility of these new constructs is somewhat in question, however, given that expression levels are reduced by 10-fold.

----------

13th Jul 2020

Dear David,

Please find below the guidelines for final submission of your Nature Structural & Molecular Biology manuscript, "Structure-guided covalent stabilization of coronavirus spike glycoprotein trimers in the closed conformation" (NSMB-A43465A). Please follow the instructions provided here and in the attached files (forms sent separately), as the formal acceptance of your manuscript will be delayed if

these issues are not addressed.

When you upload your final materials, please include a concise point-by-point response to the points below.

POLICY ISSUES:

1. Data availability: this journal strongly supports public availability of data. Please place the data used in your paper into a public data repository, or alternatively, present the data as supplementary information. If data can only be shared on request, please explain why in your Data Availability Statement, and also in the correspondence with your editor. Please note that for some data types, deposition in a public repository is mandatory.

2. DATA DEPOSITION: We require deposition of coordinates (and structure factors) into the Protein Data Bank with the designation of immediate release upon publication (HPUB). EM maps must be deposited in the EMDB. Make sure those entries are updated for immediate release.

3. Nature Research is taking an active approach to improving our transparency standards and increasing the reproducibility of all of our published results. Detailed information on experimental design and reagents is now collected on our Life Sciences Reporting Summary, which will be published alongside your paper. Please provide an updated version of the Reporting Summary (which will be published with the paper) with your final files.
https://www.nature.com/authors/policies/ReportingSummary.pdf

Please also upload a revised Editorial policy checklist; we do not need the PDB validation reports again. https://www.nature.com/authors/policies/Policy.pdf

GENERAL FORMATTING:

4. Main text, title and abstract are all fine, please see comments in attached. Online methods section also fine.

5. References: the current manuscript has 72 references in main text and 14 in methods. This is a bit above our usual limits (up to 60 references in main text; additional 20 references for online Methods), but given the high volume of recent work on the topic, we can keep them as is. Please make sure all references are cited in numerical order.

6. References: the reference list should contain papers that have been published or accepted by a named publication or recognized preprint server. Published conference abstracts, numbered patents and research datasets that have been assigned a digital object identifier may also be included in the reference list.

FIGURES AND TABLES:

7. There are currently 5 Figures and 1 Table in main article. That's fine.

8. Please make sure all figures and tables, including Extended Data Figures, are cited in the text in numerical order.

9. When submitting the revised version of your manuscript, please pay close attention to our href="https://www.nature.com/nature-research/editorial-policies/image-integrity">Digital Image Integrity Guidelines.</a> and to the following points below:
-- that unprocessed scans are clearly labelled and match the gels and western blots presented in figures.
-- that control panels for gels and western blots are appropriately described as loading or sample processing controls
-- that all images in the paper are checked for duplication of panels and for splicing of gel lanes.

Finally, please ensure that you retain unprocessed data and metadata files after publication, ideally archiving data in perpetuity, as these may be requested during the production process or after publication if any issues arise.

SUPPLEMENTARY INFORMATION

All Supplementary Information must be submitted in accordance with the instructions in the attached Inventory of Supporting Information, and should fit into one of three categories:

1. EXTENDED DATA FIGURES: you have 3 Extended Data Figures, they should be listed in section 1 of the inventory, along with their legend text.

Extended Data Figures are an integral part of the paper and only data that directly contribute to the main message should be presented. These figures will be integrated into the full-text HTML version of your paper and will be appended to the online PDF. There is a limit of 10 Extended Data Figures, and each must be referred to in the main text. Each Extended Data Figure should be of the same quality as the main figures, and should be supplied at a size that will allow both the figure and legend to be presented on a single legal-sized page. Each figure should be submitted as an individual .jpg, .tif or .eps file with a maximum size of 10 MB each. All Extended Data figure legends must be provided in the attached Inventory of Accessory Information, not in the figure files themselves.
All Extended Data Figure must be called out in order as Extended Data Fig. 1, Extended Data Fig 2, etc.

2. SUPPLEMENTARY INFORMATION: no current items (other than Reporting Summary) but we do encourage you to provide data behind graphs; that can be done as Source Data (item 3 below) or aggregated into a single table (Supplementary Table, Excel format, to be listed in section 2B of inventory)

Supplementary Information is material that is essential background to the study but which is not practical to include in the printed version of the paper (for example, large figures, video files, large data sets and calculations). Each item must be referred to in the main manuscript and detailed in the attached Inventory of Accessory Information. Supplementary Tables containing large data sets should

be in Excel format, with the table number and title included within the body of the table. All textual information and any additional Supplementary Figures (which should be presented with the legends directly below each figure) should be provided as a single, combined PDF. Please note that we cannot accept resupplies of Supplementary Information after the paper has been formally accepted unless there has been a critical scientific error.

Supplementary items (such as Supplementary Tables, Videos, Notes, and additional Supplementary Figures if permitted), should be numbered and called out in main article, as Supplementary Figure 1 (not SI1) and so on.

3. SOURCE DATA:
We encourage you to provide source data for your figures. Full-length, unprocessed gels and blots must be provided as source data for any relevant figures, and should be provided as individual PDF files for each figure containing all supporting blots and/or gels with the linked figure noted directly in the file. Statistical source data (i.e., data behind graphs) should be provided in Excel format, one file for each relevant figure, with the linked figure noted directly in the file. For imaging source data, we encourage deposition to a relevant repository, such as figshare (https://figshare.com/) or the Image Data Resource (https://idr.openmicroscopy.org).

Source data should be cited in the legend text (e.g., “Uncropped images for panels a-c are available as source data” or “Data for graphs in d-f are available as source data”).

STATISTICS and REPRODUCIBILITY
10. GRAPHS: make sure all error bars are defined (e.g., as s.d. or s.e.m.) together with a measure of center (e.g., mean or median) and precise n number and defining the type of repeat. See notes in attached.

11. When representative experiments are shown, you should state in the legends how many times each experiment was repeated independently with similar results. Please indicate number of times experiments were repeated, see notes in attached.

12. Competing interests statement: Please include a competing interests statement as a separate section after the Author Contributions, under the heading "Competing interests”, and enumerate any such circumstances there, or read: The authors declare no competing interests.

13. Reporting Summary statement: This should be placed after Online Methods section and read: Further information on experimental design is available in the Nature Research Reporting Summary linked to this article.

14. Data Availability statement: This should be placed after Reporting Summary statement (before Methods-only references). We suggest that you list in this order:
- data deposited in public repositories, with accession codes or DOIs.
- data available as Source Data (e.g. “Source data are available with the paper online.”)
- if any data can only be shared upon request, please specify what those data are and explain why.
More information and examples can be found at
http://www.nature.com/authors/policies/data/data-availability-statements-data-citations.pdf

TRANSPARENT PEER REVIEW
15. Nature Structural & Molecular Biology offers a transparent peer review option for new original research manuscripts submitted from 1st December 2019. We encourage increased transparency in peer review by publishing the reviewer comments, author rebuttal letters and editorial decision letters if the authors agree. Such peer review material is made available as a supplementary peer review file. <b>Please state in the cover letter 'I wish to participate in transparent peer review' if you want to opt in, or 'I do not wish to participate in transparent peer review' if you don't.</b> Failure to state your preference will result in delays in accepting your manuscript for publication.
Please note: we allow redactions to authors' rebuttal and reviewer comments in the interest of confidentiality. If you are concerned about the release of confidential data, please let us know specifically what information you would like to have removed. Please note that we cannot incorporate redactions for any other reasons. Reviewer names will be published in the peer review files if the reviewer signed the comments to authors, or if reviewers explicitly agree to release their name. For more information, please refer to our <a href="https://www.nature.com/documents/nr-transparent-peer-review.pdf" target="new">FAQ page</a>.

AUTHORSHIP AND OTHER REQUIREMENTS

16. Ensure that all required forms found in the Policy Worksheet are uploaded to our Journal Processing system as "Supplementary Materials".

17. <b>ORCID</b>: your account is already linked to your ORCID; we encourage all coauthors to do the same.
https://www.springernature.com/gp/researchers/orcid/orcid-for-nature-research

Nature Structural & Molecular Biology is committed to improving transparency in authorship. As part of our efforts in this direction, we are now requesting that all authors identified as 'corresponding author' create and link their Open Researcher and Contributor Identifier (ORCID) with their account on the Manuscript Tracking System (MTS) prior to acceptance. ORCID helps the scientific community achieve unambiguous attribution of all scholarly contributions. For more information please visit http://www.springernature.com/orcid

For all corresponding authors listed on the manuscript, please follow the instructions in the link below to link your ORCID to your account on our MTS before submitting the final version of the manuscript. If you do not yet have an ORCID you will be able to create one in minutes.
https://www.springernature.com/gp/researchers/orcid/orcid-for-nature-research

IMPORTANT: All authors identified as 'corresponding author' on the manuscript must follow these instructions. Non-corresponding authors do not have to link their ORCIDs but are encouraged to do so. Please note that it will not be possible to add/modify ORCIDs at proof. Thus, if they wish to have their ORCID added to the paper they must also follow the above procedure prior to acceptance.

To support ORCID's aims, we only allow a single ORCID identifier to be attached to one account. If you have any issues attaching an ORCID identifier to your MTS account, please contact the <a href=""http://platformsupport.nature.com/"">Platform Support Helpdesk</a>.

Nature Research journals <a href="https://www.nature.com/nature-research/editorial-policies/reporting-standards#protocols" target="new">encourage authors to share their step-by-step experimental protocols</a> on a protocol sharing platform of their choice. Nature Research's Protocol Exchange is a free-to-use and open resource for protocols; protocols deposited in Protocol Exchange are citable and can be linked from the published article. More details can found at <a href="https://www.nature.com/protocolexchange/about" target="new">www.nature.com/protocolexchange/about</a>.

In addition to addressing these points, please refer to the attached policy and rights worksheet, which contains information on how to comply with our legal guidelines for publication and describes the files that you will need to upload prior to final acceptance. You must initial the relevant portions of this checklist, sign it and return it with your final files. I have also attached a formatting guide for you to consult as you prepare the revised manuscript. Careful attention to this guide will ensure that the production process for your paper is more efficient.

Please use the following link for uploading these materials:
[REDACTED]

We ask that you aim to return your revised paper within 7 days. If you have any further questions, please feel free to contact me.

Best regards,

Ines Chen, Ph.D.
Chief Editor
Nature Structural & Molecular Biology

ORCID 0000-0002-1405-9703

Reviewer #1:
Remarks to the Author:
The authors have responded to suggestions from reviewers, and I think the article would now be suitable for publication in NSMB. The utility of these new constructs is somewhat in question, however, given that expression levels are reduced by 10-fold.

**Author Rebuttal, first revision:**

Dear Nyx,

I agree to participate in transparent peer-review.

Best regards

David Veesler, PhD
Associate Professor
Department of Biochemistry
University of Washington, Seattle

http://faculty.washington.edu/dveesler/
@veeslerlab

**Final Decision Letter:**

20th Jul 2020

Dear David,

We are now happy to accept your revised paper "Structure-guided covalent stabilization of coronavirus spike glycoprotein trimers in the closed conformation" for publication as a Article in Nature Structural & Molecular Biology.

Acceptance is conditional on the manuscript's not being published elsewhere and on there being no announcement of this work to the newspapers, magazines, radio or television until the publication date in Nature Structural & Molecular Biology.

Before the manuscript is sent to the printers, we shall make any detailed changes in the text that may be necessary either to make it conform with house style or to make it intelligible to a wider readership. If the changes are extensive, we will ask for your approval before the manuscript is laid out for production. Once your manuscript is typeset you will receive a link to your electronic proof via email within 20 working days, with a request to make any corrections within 48 hours. Please read proofs with great care to make sure that the sense has not been altered. If you have queries at any point during the production process then please contact the production team at rjsproduction@springernature.com. Once your paper has been scheduled for online publication, the Nature press office will be in touch to confirm the details.

Please note that due to tight production schedules, proofs should be returned as quickly as possible to avoid delaying publication. If you anticipate any limitations to your availability over the next 2-4 weeks (such as vacation or traveling to conferences, etc.), please e-mail rjsproduction@springernature.com as soon as possible. Please provide specific dates that you will be unavailable and provide detailed contact information for an alternate corresponding author if

necessary.

To assist our authors in disseminating their research to the broader community, our SharedIt initiative provides all co-authors with the ability to generate a unique shareable link that will allow anyone (with or without a subscription) to read the published article. Recipients of the link with a subscription will also be able to download and print the PDF.

As soon as your article is published, you can generate your shareable link by entering the DOI of your article here: <a href="http://authors.springernature.com/share">http://authors.springernature.com/share<a>. Corresponding authors will also receive an automated email with the shareable link

Note the policy of the journal on data deposition: http://www.nature.com/authors/policies/availability.html.

Your paper will be published online soon after we receive proof corrections and will appear in print in the next available issue. You can find out your date of online publication by contacting the production team shortly after sending your proof corrections. The embargo is set at 16:00 London time (GMT) / 11:00 am US Eastern time (EST), on the Monday of publication. Now is the time to inform your Public Relations or Press Office about your paper, as they might be interested in promoting its publication. This will allow them time to prepare an accurate and satisfactory press release. Include your manuscript tracking number (NSMB-A43465B) and our journal name, which they will need when they contact our press office.

About one week before your paper is published online, we shall be distributing a press release to news organizations worldwide, which may very well include details of your work. We are happy for your institution or funding agency to prepare its own press release, but it must mention the embargo date and Nature Structural & Molecular Biology. If you or your Press Office have any enquiries in the meantime, please contact press@nature.com.

You can now use a single sign-on for all your accounts, view the status of all your manuscript submissions and reviews, access usage statistics for your published articles and download a record of your refereeing activity for the Nature journals.

If you have not already done so, we strongly recommend that you upload the step-by-step protocols used in this manuscript to the Protocol Exchange. Protocol Exchange is an open online resource that allows researchers to share their detailed experimental know-how. All uploaded protocols are made freely available, assigned DOIs for ease of citation and fully searchable through nature.com. Protocols can be linked to any publications in which they are used and will be linked to from your article. You can also establish a dedicated page to collect all your lab Protocols. By uploading your Protocols to Protocol Exchange, you are enabling researchers to more readily reproduce or adapt the methodology you use, as well as increasing the visibility of your protocols and papers. Upload your Protocols at www.nature.com/protocolexchange/. Further information can be found at www.nature.com/protocolexchange/about.

An online order form for reprints of your paper is available at <a href="https://www.nature.com/reprints/author-reprints.html">https://www.nature.com/reprints/author-reprints.html</a>. Please let your coauthors

and your institutions' public affairs office know that they are also welcome to order reprints by this method.

Please note that we encourage the authors to self-archive their manuscript (the accepted version before copy editing) in their institutional repository, and in their funders' archives, six months after publication. Nature Research Group recognizes the efforts of funding bodies to increase access of the research they fund, and strongly encourages authors to participate in such efforts. For information about our editorial policy, including license agreement and author copyright, please visit www.nature.com/nsmb/ about/ed_policies/index.html

The Author's Accepted Manuscript (the accepted version of the manuscript as submitted by the author) may only be posted 6 months after the paper is published, consistent with our <a href="http://www.nature.com/authors/policies/license.html">self-archiving embargo</a>. Please note that the Author’s Accepted Manuscript may not be released under a Creative Commons license. For Nature Research Terms of Reuse of archived manuscripts please see: <a href="http://www.nature.com/authors/policies/license.html#terms">http://www.nature.com/authors/policies/license.html#terms</a>
If you have posted a preprint on any preprint server, please ensure that the preprint details are updated with a publication reference, including the DOI and a URL to the published version of the article on the journal website.

Sincerely,
Ines

Ines Chen, Ph.D.
Chief Editor
Nature Structural & Molecular Biology

ORCID 0000-0002-1405-9703

Click here if you would like to recommend Nature Structural & Molecular Biology to your librarian: http://www.nature.com/subscriptions/recommend.html#forms
